# Clinical Impact of Telemedicine Interventions on Outcomes After Abdominal Surgery: A Systematic Review of Randomized Controlled Trials

**DOI:** 10.7759/cureus.90236

**Published:** 2025-08-16

**Authors:** Mohammed Awad Siddig Mohammed, Lobaba Mubarak Saidahmed Ahmed, Ahmed Mohamed Elamin Mubarak Osman, Esra Eltahir Mohamed Gamareldin, Aseel Khalid Ahmed Ibrahim, Hanady ME M Osman

**Affiliations:** 1 General Surgery, Damad General Hospital, Ministry of Health, Jizan, SAU; 2 Surgery, Shendi University, Shendi, SDN; 3 General Medicine, Jouf University Medical Services Center, Sakaka, SAU; 4 General Practice, Faculty of Medicine, Assiut University, Assiut, EGY; 5 Quality Improvement and Patient Safety, Najran Armed Forces Hospital, Ministry of Defense Health Services, Najran, SAU

**Keywords:** abdominal surgery, digital health, patient outcomes, postoperative care, randomized controlled trials, telemedicine

## Abstract

Abdominal surgery is associated with significant postoperative morbidity and healthcare utilization, prompting the exploration of telemedicine as a tool to improve outcomes. While previous reviews have examined telemedicine in specific surgical contexts, this systematic review provides a comprehensive synthesis of randomized controlled trials (RCTs) across diverse abdominal procedures. This systematic review evaluates the clinical impact of telemedicine interventions on postoperative outcomes, including morbidity, patient satisfaction, readmissions, and cost-effectiveness. Following Preferred Reporting Items for Systematic Reviews and Meta-Analyses (PRISMA) guidelines, we conducted a systematic search of PubMed, Scopus, Web of Science, and ClinicalTrials.gov for RCTs evaluating telemedicine in abdominal surgery. Eligible studies reported clinical outcomes such as recovery time, readmissions, and patient satisfaction. Two reviewers independently screened records, extracted data, and assessed risk of bias using the Cochrane Risk of Bias 2 (RoB 2) tool. Due to heterogeneity, findings were synthesized narratively. The review included 12 RCTs encompassing various abdominal surgeries and telemedicine approaches. Results demonstrated that telemedicine interventions consistently improved patient satisfaction and showed noninferiority compared to traditional in-person follow-up. Several studies reported reduced recovery times and improved postoperative monitoring through remote technologies. However, effects on hospital readmissions were mixed, with some interventions showing benefit while others demonstrated no significant difference. Cost-effectiveness analyses indicated potential savings from smartphone-based follow-up systems. Most studies exhibited low risk of bias, though some limitations were noted regarding unblinded designs and subjective outcome measures. These findings suggest that telemedicine offers valuable opportunities to enhance postoperative care after abdominal surgery, particularly in improving patient-centered outcomes and recovery experiences. The variable impact on clinical outcomes like readmissions highlights the importance of tailoring interventions to specific surgical contexts and patient needs. Future research should focus on standardizing outcome measures, evaluating long-term effects, and addressing implementation challenges to maximize the potential of telemedicine in surgical care.

## Introduction and background

Abdominal surgery encompasses a wide range of procedures, from minimally invasive laparoscopic interventions to complex open surgeries, each associated with varying degrees of postoperative morbidity, recovery time, and healthcare resource utilization [1]. Despite advancements in surgical techniques and perioperative care, postoperative complications, such as surgical site infections, ileus, and cardiopulmonary events, remain significant challenges, often leading to prolonged hospital stays, increased healthcare costs, and reduced patient satisfaction [2]. Effective postoperative monitoring and timely intervention are crucial to improving clinical outcomes, yet traditional in-person follow-up may be limited by geographical barriers, patient mobility issues, and healthcare system constraints [3].

Telemedicine, defined as the remote delivery of healthcare services using telecommunications technology, has emerged as a transformative approach to postoperative care [4]. By enabling virtual consultations, remote vital sign monitoring, and digital patient-reported outcome assessments, telemedicine has the potential to enhance patient accessibility, facilitate early detection of complications, and reduce unnecessary hospital readmissions [5]. Recent studies suggest that telemedicine interventions may improve patient adherence to postoperative instructions, decrease emergency department visits, and shorten recovery times [3,6]. However, the extent of its clinical impact, particularly on hard endpoints such as mortality, complication rates, and hospital length of stay (LOS), remains uncertain due to heterogeneous study designs and varying intervention protocols [7].

While previous reviews have explored telemedicine in surgical care, most have focused on specific procedures (e.g., bariatric or colorectal surgery) or non-randomized studies, which are susceptible to bias. A systematic synthesis of randomized controlled trials (RCTs), the gold standard for evaluating clinical interventions, is needed to provide robust evidence on the efficacy of telemedicine in improving outcomes after abdominal surgery. This systematic review aims to critically evaluate the clinical impact of telemedicine interventions on postoperative outcomes, including morbidity, mortality, hospital readmission rates, patient satisfaction, and cost-effectiveness, by analyzing data from RCTs. By doing so, this review will inform clinical practice, guide future research, and highlight gaps in the current evidence base.

## Review

Methodology

Review Design and Protocol

This systematic review was conducted in accordance with the Preferred Reporting Items for Systematic Reviews and Meta-Analyses (PRISMA) guidelines to ensure methodological rigor and transparency [8]. The following sections outline the key steps taken, including search strategy, study selection, data extraction, risk of bias assessment, and data synthesis.

Eligibility Criteria

Studies were included if they met the following criteria: (1) RCTs evaluating telemedicine interventions in patients undergoing abdominal surgery; (2) studies reporting clinical outcomes such as readmission rates, patient satisfaction, recovery time, and cost-effectiveness; and (3) full-text articles published in English. Exclusion criteria comprised non-RCTs (e.g., observational studies, reviews), studies not focused on telemedicine, and those lacking comparative data between intervention and control groups.

Information Sources and Search Strategy

A comprehensive literature search was performed across multiple databases, including PubMed, Scopus, Web of Science, and ClinicalTrials.gov. The search strategy combined Medical Subject Headings (MeSH) terms and keywords related to telemedicine, abdominal surgery, postoperative outcomes, and RCTs. Additional studies were identified through citation searching and manual screening of reference lists from relevant reviews.

Study Selection Process

**Figure 1:**
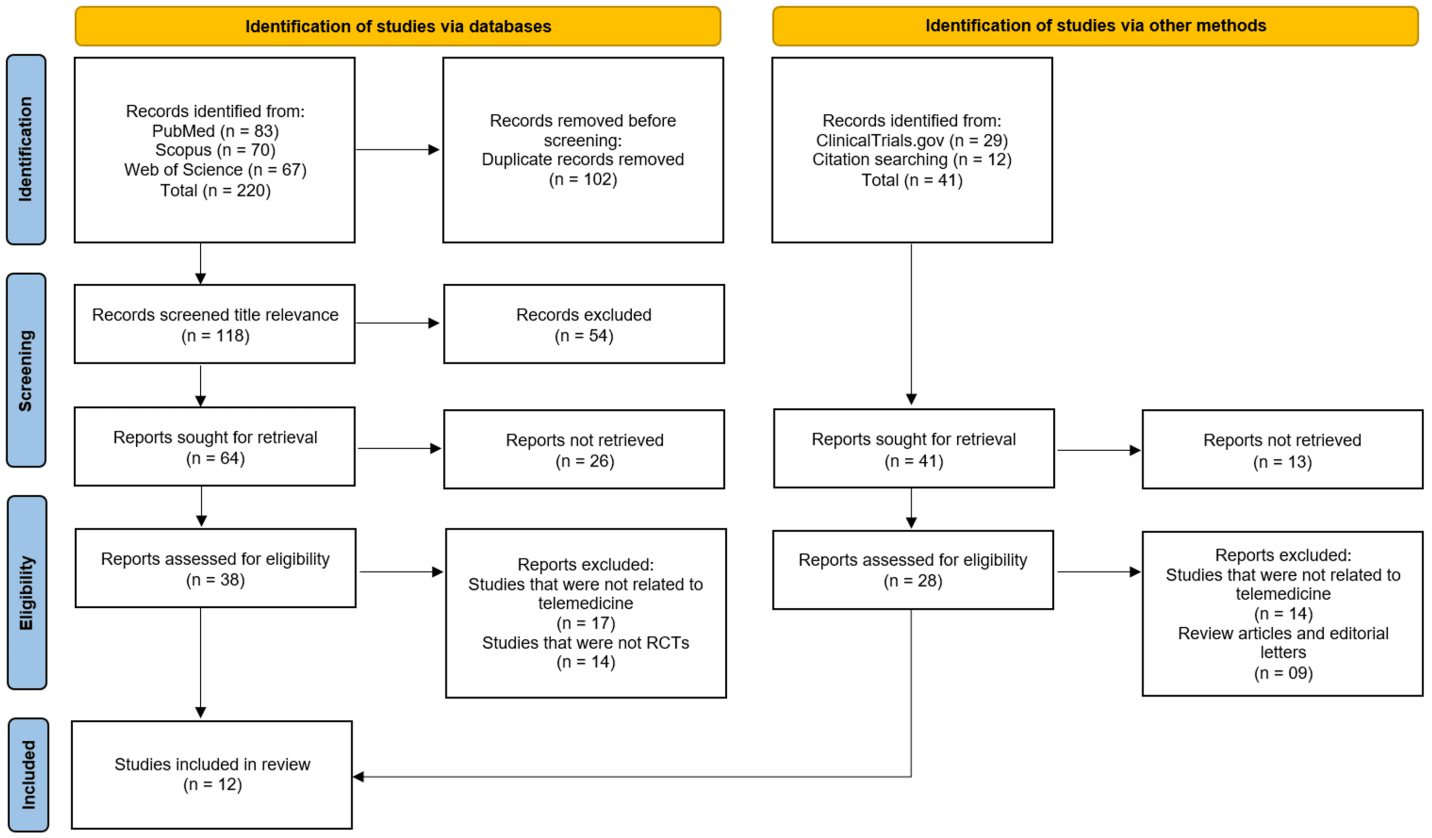
Study Selection Process According to PRISMA 2020 Guidelines

The study selection process followed the PRISMA flowchart (Figure 1). Initially, all identified records were imported into an EndNote X9 reference management software (Clarivate Analytics, Philadelphia, PA, USA) to remove duplicates. Two independent reviewers (MASM and HMMO), from the list of authors, screened titles and abstracts for relevance, followed by a full-text assessment of potentially eligible studies. Discrepancies were resolved through discussion or consultation with a third reviewer (AKAI).

Data Extraction

A standardized data extraction form was developed to collect key study characteristics, including author, year, country, study design, sample size, type of surgery, telemedicine intervention details, control group protocol, primary and secondary outcomes, follow-up duration, and key findings. Data extraction was performed independently by two reviewers, with cross-verification to ensure accuracy.

Risk of Bias Assessment

The Cochrane Risk of Bias 2 (RoB 2) tool was used to evaluate the methodological quality of included RCTs [9]. Each study was assessed across five domains: (1) randomization process, (2) deviations from intended interventions, (3) missing outcome data, (4) measurement of outcomes, and (5) selection of reported results. Studies were categorized as having low risk, some concerns, and high risk of bias. Discrepancies in assessments were resolved through consensus.

Data Synthesis

Due to heterogeneity in interventions, surgical procedures, and outcome measures, a meta-analysis was not feasible. Instead, findings were synthesized narratively, with outcomes categorized into themes such as patient satisfaction, recovery time, readmission rates, and cost-effectiveness. Comparative analysis was performed to identify patterns and discrepancies across studies.

Ethical Considerations

As this study involved analysis of previously published data, ethical approval was not required. However, all included studies were reviewed for compliance with ethical standards, including informed consent and institutional review board approval where applicable.

Results

Search Results

The systematic review followed the PRISMA guidelines to identify relevant studies. A total of 261 records were initially identified through database searches (PubMed: n = 83; Scopus: n = 70; Web of Science: n = 67) and additional methods (ClinicalTrials.gov: n = 29; citation searching: n = 12). After removing 102 duplicate records, 118 studies underwent title and abstract screening, of which 54 were excluded due to irrelevance. Subsequently, 105 full-text reports were sought for retrieval, but 39 were unavailable, leaving 66 reports for eligibility assessment. Of these, 40 were excluded for the following reasons: studies unrelated to telemedicine (n = 31), review articles/editorials (n = 9), and non-randomized controlled trials (n = 14). Ultimately, 12 studies [10-21] met the inclusion criteria and were incorporated into the systematic review (Figure 1).

Study Characteristics

The systematic review included 12 RCTs [10-21] evaluating the clinical impact of telemedicine interventions on outcomes after abdominal surgery. The studies were conducted across diverse geographical regions, including Australia, the Netherlands, USA, Canada, Sweden, and Spain, and encompassed a wide range of abdominal surgeries such as colorectal cancer resection, laparoscopic cholecystectomy, pelvic floor surgery, and day-case procedures (Table 1). Sample sizes varied significantly, ranging from small cohorts (e.g., 26 patients per arm in Lee et al.'s study [16]) to larger multicenter trials (e.g., 997 patients in Jaensson et al.'s study [17]). Telemedicine interventions included centralized nurse-led telephone follow-ups, smartphone-based recovery apps, virtual clinical encounters, and remote automated monitoring (RAM) systems. Control groups typically received standard postoperative care, including in-person visits or paper-based follow-up.

**Table 1:** Key characteristics and findings of studies included in the review ED: emergency department; QOL: quality of life; S-CAHPS: Consumer Assessment of Healthcare Providers and Systems - Surgical Care; PFDI-20: Pelvic Floor Distress Inventory-20; RAM: remote automated monitoring; ERP: enhanced recovery pathway; GI: gastrointestinal; RAPP: recovery assessment by phone points; SwQoR questionnaire: Swedish web version of the quality of recovery questionnaire; QALY: quality-adjusted life year; POD: postoperative day; LOS: length of stay; NR: not reported

Author (Year)	Country	Study Design	Sample Size (Intervention/Control)	Type of Abdominal Surgery	Telemedicine Intervention	Control Group	Primary Outcomes Measured	Follow-Up Duration	Key Findings
Young et al. (2013) [10]	Australia	RCT	387/369	Colorectal cancer surgery	Centralized, nurse-delivered standardized telephone calls at 3 and 10 days, and 1, 3, and 6 months post-discharge	Usual care	Unmet supportive care needs, care coordination experience, unplanned readmissions, ED presentations, distress, QOL	6 months	No significant differences between groups in any primary outcomes. Intervention did not improve postoperative outcomes. Tailored interventions may be needed.
Van Der Meij et al. (2018) [11]	Netherlands	Multicentre, single-blind RCT	173/171	Laparoscopic cholecystectomy, inguinal hernia surgery, laparoscopic adnexal surgery (benign)	Personalised perioperative e-health programme providing tailored recovery guidance and expectation management	Usual care + placebo website with general recovery advice	Time to return to normal activities	6 months	Median return to normal activities was significantly shorter in the intervention group; no difference in complications
Thompson et al. (2019) [12]	USA	RCT	50/50	Pelvic floor surgery	Telephone follow-up calls by a nurse at 2, 6, and 12 weeks postoperatively	In-person clinic visits at 2, 6, and 12 weeks postoperatively	Patient satisfaction (S-CAHPS), safety, clinical outcomes (PFDI-20, pain scales)	3 months	Telephone follow-up was noninferior in patient satisfaction; no difference in clinical outcomes or adverse events; reduced patient/provider burden for postoperative care
Pooni et al. (2023) [13]	Canada	RCT	282	Elective colorectal surgery	Postdischarge monitoring via mobile app + usual care	Usual postoperative care	30-day readmissions, ER visits, primary care visits, unplanned healthcare visits, patient-reported outcomes	30 days	No significant difference in readmissions or ER visits; significant improvements in patient-reported satisfaction, well-being, and reduced anxiety with app use
McGillion et al. (2021) [14]	Canada	Multicentre RCT	451/454	Non-elective abdominal surgery	Virtual care with RAM via tablet (daily vitals + wound photos + nurse interaction)	Standard post-discharge care	Days alive at home	31 days	No significant difference in days alive at home. The RAM group had fewer hospital readmissions, more drug errors detected and corrected, and less pain at days 7, 15, and 30
Mata et al. (2020) [15]	Canada	RCT (assessor-blind, sham-controlled)	49/48	Colorectal resection	iPad with a novel mobile device app for postoperative education and self-assessment	iPad without the app	Mean adherence (%) to five ERP elements: mobilization, GI motility stimulation, breathing exercises, oral liquid intake, and nutritional drink intake	First 2 postoperative days	No significant difference in adherence between groups; the mobile app did not improve ERP adherence.
Lee et al. (2021) [16]	USA	Randomized controlled noninferiority trial	26/26	Reconstructive surgery for pelvic organ prolapse	Virtual clinical encounters via video conference technology	Traditional in-office clinical encounters	Patient satisfaction (Patient Satisfaction Questionnaire-18); postoperative health care utilization; complication rates	30 days (primary outcome), up to 90 days for secondary outcomes	Virtual follow-up was noninferior to in-office visits in patient satisfaction; no significant differences in complication rates or healthcare utilization
Jaensson et al. (2017) [17]	Sweden	Single-blind, multicentre RCT	997 (RAPP/standard care)	Day surgery under anaesthesia	Smartphone-based recovery assessment using the RAPP app with the SwQoR questionnaire	Standard care with paper-based follow-up	Postoperative recovery assessed by the SwQoR questionnaire (sleep quality, pain, well-being, etc.)	14 days (assessed on postoperative days 7 and 14)	The RAPP group had significantly better recovery scores on 7 of 24 SwQoR items on day 7, including less pain, better sleep, and improved well-being; both men and women benefited equally from the intervention
Halder et al. (2022) [18]	USA	RCT	63/69	Urogynecological surgery (pelvic organ prolapse and/or stress urinary incontinence)	Preoperative provider-initiated telehealth call (3±2 days before surgery) + usual preoperative counseling	Usual preoperative counseling alone	Surgical preparedness (Preoperative Preparedness Questionnaire); various postoperative outcomes	4-8 weeks	The telehealth call group had significantly better surgical preparedness, understanding of surgery, and perception of care time; no significant differences in postoperative outcomes between groups at 4-8 weeks
Dahlberg et al. (2017) [19]	Sweden	Prospective parallel single-blind multicentre RCT	477/477	Day-case abdominal surgery	Smartphone-based app (RAPP) for postoperative recovery assessment	Standard care follow-up	Cost-effectiveness, QALYs	2 weeks	RAPP was cost-effective with €4.77 savings per patient; no significant difference in QALYs; 71% probability of being cost-effective
Bednarski et al. (2019) [20]	USA	Phase II RCT	14/16	Minimally invasive colorectal resection	TeleRecovery: accelerated discharge (POD 1) + televideo consultation on POD 2	Standard postoperative care	Total 30-day LOS	30 days	TeleRecovery reduced 30-day LOS without affecting quality of life, pain levels, or satisfaction. No difference in adverse events noted.
Cremades et al. (2020) [21]	Spain	RCT	100/100	General surgery	Telemedicine follow-up in outpatient clinics	Conventional in-person follow-up	Feasibility of telemedicine follow-up; clinical outcomes; patient satisfaction	NR	Telemedicine follow-up was feasible in 74% vs. 90% in control; no significant differences in clinical outcomes or patient satisfaction

Primary Outcomes

The primary outcomes assessed across studies were heterogeneous, reflecting the diversity of telemedicine applications. Key measures included patient satisfaction, time to return to normal activities, postoperative recovery scores, readmission rates, and cost-effectiveness. For instance, Young et al. [10] evaluated unmet supportive care needs and care coordination but found no significant differences between telemedicine and standard care groups. In contrast, Van Der Meij et al. [11] reported a statistically significant reduction in time to return to normal activities (HR = 1.38, 95% CI 1.09-1.73, p = 0.007) with a personalized e-health program. Similarly, Jaensson et al. [17] demonstrated improved postoperative recovery scores (Swedish web version of the quality of recovery, or SwQoR, questionnaire) on day 7, with significant reductions in pain and better sleep quality (p < 0.05).

Patient Satisfaction and Safety

Telemedicine interventions were consistently noninferior to standard care in terms of patient satisfaction and safety. Thompson et al. [12] found no significant differences in satisfaction scores (92% vs. 88%, p = 0.006) or adverse events (p = 0.36) between telephone follow-up and in-person visits after pelvic floor surgery. Lee et al. [16] corroborated these findings, showing noninferior satisfaction with video-based follow-up for pelvic organ prolapse surgery (difference −0.46, 95% CI −1.95 to 1.03). Notably, telemedicine was associated with reduced patient and provider burden, as highlighted by Thompson et al. [12] and Lee et al. [16].

Readmissions and Healthcare Utilization

The impact of telemedicine on readmissions and healthcare utilization was mixed. Pooni et al. [13] observed no significant reduction in 30-day readmissions or emergency room (ER) visits with a postdischarge mobile app (p = 0.55), though patient-reported outcomes improved significantly (p = 0.001). Conversely, McGillion et al. [14] reported fewer hospital readmissions and more detected/corrected drug errors (Δ ~24%) with RAM-based virtual care, despite no difference in the primary outcome of days alive at home. Bednarski et al. [20] demonstrated a reduction in 30-day LOS with a structured telemedicine program (median LOS: 28.3 vs. 51.5 hours, p = 0.041).

Cost-Effectiveness and Feasibility

Two studies evaluated cost-effectiveness, with Dahlberg et al. [19] reporting that the recovery assessment by phone points (RAPP) smartphone app was cost-effective (mean savings: €23.66 per patient, p = 0.008) without compromising quality-adjusted life years (QALYs). Cremades et al. [21] found telemedicine follow-up feasible (74% vs. 90% in controls, p = 0.003) but noted no differences in clinical outcomes or satisfaction.

Summary of Findings

Overall, telemedicine interventions showed variable effects on clinical outcomes (Table 2). While some studies reported benefits such as faster recovery [11,17], improved patient satisfaction [12,16], and cost savings [19], others found no significant differences in primary outcomes like readmissions [10,13] or enhanced recovery pathway (ERP) adherence [15]. The heterogeneity in interventions, surgical populations, and outcome measures underscores the need for tailored approaches to maximize the potential of telemedicine in abdominal surgery.

**Table 2:** Summary of clinical outcomes reported in included studies ED: emergency department; QOL: quality of life; RAM: remote automated monitoring; ERP: enhanced recovery pathway; GI: gastrointestinal; RAPP: recovery assessment by phone points; SwQoR questionnaire: Swedish web version of the quality of recovery questionnaire; QALY: quality-adjusted life year; POD: postoperative day; LOS: length of stay

Author (Year)	Telemedicine Intervention	Primary Outcome(s)	Effect Size/Statistical Significance	Direction of Effect
Young et al. (2013) [10]	Centralized, nurse-delivered telephone-based follow-up at 3 and 10 days, and 1, 3, and 6 months post-discharge	Unmet supportive care needs, care coordination experience, unplanned readmissions, ED visits, distress, QOL	No statistically significant differences: ED visits (10.8% vs. 13.8%; p = 0.2); readmissions at 1 month (8.6% vs. 10.5%; p = 0.4); readmissions at 6 months (25.6% vs. 27.9%; p = 0.5); no differences in other outcomes	No effect observed/neutral
Van Der Meij et al. (2018) [11]	Personalised perioperative e-healthcare programme	Time to return to normal activities	HR = 1.38, 95% CI 1.09–1.73, p = 0.007	Faster return to normal activities in the intervention group
Thompson et al. (2019) [12]	Telephone follow-up at 2, 6, and 12 weeks postoperatively	Patient satisfaction, adverse events, clinical outcomes	Satisfaction noninferior (92% vs. 88%, p = 0.006); no significant differences in adverse events (p = 0.36) or clinical outcomes (p > 0.05)	Noninferior satisfaction; no difference in safety or clinical outcomes
Pooni et al. (2023) [13]	Mobile app for post-discharge monitoring after colorectal surgery	30-day readmission, ER visits, patient-reported outcomes	No significant difference in readmission (p = 0.55) or ER visits; improved satisfaction, well-being, and anxiety (all p = 0.001)	No effect on readmissions; positive effect on patient-reported outcomes
McGillion et al. (2021) [14]	Virtual care with RAM	Days alive at home; acute hospital care; drug error detection/correction; pain at days 7, 15, 30	No significant difference in days alive at home (RR 1.01); reduced acute care use (RR 0.80); significantly more drug errors detected/corrected (Δ ~24%); significantly less pain (Δ 9.6–13.9%)	Mixed: no effect on primary outcome; favorable effects on secondary outcomes
Mata et al. (2020) [15]	Mobile device app for postoperative education and self-assessment (via iPad)	Mean adherence (%) to five postoperative ERP elements (mobilization, GI motility stimulation, breathing exercises, oral liquids, nutritional drinks)	Adjusted mean difference: 2.4% (95% CI −5 to 10%), p = 0.53	No significant effect; adherence similar in both groups
Lee et al. (2021) [16]	Video conference follow-up after pelvic organ prolapse surgery	Patient satisfaction, postoperative complications, healthcare utilization	Satisfaction score difference −0.46 (95% CI −1.95 to 1.03); p > 0.05, for all secondary outcomes	Noninferior satisfaction; no significant differences in complications or utilization
Jaensson et al. (2017) [17]	Smartphone-based recovery assessment (RAPP)	Postoperative recovery assessed via SwQoR on postoperative days 7 and 14	Statistically significant improvements in 7 of 24 SwQoR items on day 7 (p < 0.05)	Improved postoperative recovery; reduced discomfort and symptoms
Halder et al. (2022) [18]	Preoperative telehealth call (3±2 days before surgery) plus usual counseling	Surgical preparedness	82.5% vs. 59.4%, p < 0.01	Significantly improved preparedness; no difference in postoperative outcomes
Dahlberg et al. (2017) [19]	RAPP – smartphone-based follow-up after day surgery	Cost-effectiveness (healthcare costs, QALYs)	Mean cost difference: €23.66 (99% CI −46.57 to −0.76; p = 0.008). No significant difference in QALYs (p = 0.75)	Cost: favoured intervention (RAPP); QALY: no difference
Bednarski et al. (2019) [20]	Structured telemedicine program (TeleRecovery) with POD 2 televideoconference	30-day total LOS	Median LOS: 28.3 h vs. 51.5 h; p = 0.041	Reduced LOS
Cremades et al. (2020) [21]	Telemedicine follow-up after general surgery	Feasibility, clinical outcomes, patient satisfaction	Feasibility lower in telemedicine (74% vs. 90%, p = 0.003); no difference in clinical outcomes (p = 0.832) or satisfaction (p = 0.099)	Mixed: lower feasibility, no difference in clinical outcomes or satisfaction

Results of Risk of Bias

The risk of bias assessment using the Cochrane ROB 2 tool revealed that the majority of the included studies demonstrated a low risk of bias across all domains, including randomization process, deviations from intended interventions, missing outcome data, measurement of outcomes, and selection of reported results [10-13, 16-20]. However, some concerns were identified in a subset of studies. McGillion et al. [14] and Mata et al. [15] raised some concerns due to potential biases in deviations from intended interventions (e.g., unblinded designs) and measurement of outcomes (e.g., self-reported adherence or recovery metrics). Similarly, Cremades et al. [21] had some concerns regarding feasibility assessment and subjective satisfaction measures. Notably, no studies were judged to have a high risk of bias, underscoring the overall methodological robustness of the evidence (Table 3).

**Table 3:** Risk of bias assessment using the Cochrane Risk of Bias 2 (RoB 2) tool

Study (Author, Year)	Randomization Process	Deviations from Intended Interventions	Missing Outcome Data	Measurement of Outcomes	Selection of Reported Results	Overall Risk of Bias
Young et al. (2013) [10]	Low	Low	Low	Low	Low	Low
Van Der Meij et al. (2018) [11]	Low	Low	Low	Low	Low	Low
Thompson et al. (2019) [12]	Low	Low	Low	Low	Low	Low
Pooni et al. (2023) [13]	Low	Low	Low	Low	Low	Low
McGillion et al. (2021) [14]	Low	Some concerns	Low	Some concerns	Low	Some concerns
Mata et al. (2020) [15]	Low	Some concerns	Low	Some concerns	Low	Some concerns
Lee et al. (2021) [16]	Low	Low	Low	Low	Low	Low
Jaensson et al. (2017) [17]	Low	Low	Low	Low	Low	Low
Halder et al. (2022) [18]	Low	Low	Low	Low	Low	Low
Dahlberg et al. (2017) [19]	Low	Low	Low	Low	Low	Low
Bednarski et al. (2019) [20]	Low	Low	Low	Low	Low	Low
Cremades et al. (2020) [21]	Low	Some concerns	Low	Some concerns	Low	Some concerns

Discussion

The findings of this systematic review highlight the diverse clinical impacts of telemedicine interventions on outcomes following abdominal surgery, synthesizing evidence from 12 RCTs [10-21]. The results demonstrate that while telemedicine shows promise in certain domains, such as improving patient satisfaction, reducing time to recovery, and enhancing cost-effectiveness, its effects on other critical outcomes, like readmission rates and adherence to enhanced recovery protocols, remain inconsistent. These observations align with broader trends in the telemedicine literature, where the heterogeneity of interventions, patient populations, and outcome measures often leads to variable results. For instance, the study by Van Der Meij et al. [11] reported a significant reduction in time to return to normal activities (HR = 1.38, p = 0.007) using a personalized e-health program, a finding that resonates with prior research emphasizing the role of tailored postoperative guidance in accelerating recovery. Similarly, Jaensson et al. [17] demonstrated that a smartphone-based recovery app (RAPP) improved postoperative recovery scores, particularly in relation to pain and sleep quality, which echoes the growing body of evidence supporting digital tools for symptom monitoring and patient engagement. However, the lack of significant improvements in readmission rates in studies like those by Young et al. [10] and Pooni et al. [13] suggests that telemedicine may not uniformly address all postoperative challenges, particularly those requiring hands-on clinical intervention.

Patient satisfaction emerged as a consistent strength of telemedicine interventions across multiple studies, reinforcing its utility as a viable alternative to traditional in-person follow-up. Thompson et al. [12] and Lee et al. [16] both found that telemedicine was noninferior to standard care in terms of satisfaction, with the added benefit of reducing patient and provider burden. These findings are corroborated by a meta-analysis by Monaghesh and Hajizadeh [22], which highlighted high patient acceptance of telemedicine in surgical follow-ups, particularly for routine postoperative assessments. The convenience of virtual encounters, as demonstrated by Lee et al. [16], may be especially advantageous for patients in remote areas or those with mobility limitations, a point further supported by broader health equity discussions in telemedicine adoption. However, the study by Cremades et al. [21] noted lower feasibility (74% vs. 90%) for telemedicine follow-up, suggesting that technological barriers or patient preferences may limit its universal applicability. This aligns with the existing literature emphasizing the "digital divide," where disparities in access to technology or digital literacy can hinder the effectiveness of telemedicine interventions, particularly among older or socioeconomically disadvantaged populations.

The mixed results regarding healthcare utilization, such as readmissions and emergency visits, warrant careful interpretation. While McGillion et al. [14] reported fewer hospital readmissions and improved detection of drug errors with RAM, Pooni et al. [13] found no significant reduction in 30-day readmissions despite improvements in patient-reported outcomes. This discrepancy may reflect differences in intervention design; for example, RAM’s real-time vital monitoring and nurse interaction [14] may address complications more proactively than a standalone mobile app [13]. These findings parallel a systematic review by Eysenbach [23], which noted that telemedicine interventions with active clinician involvement tend to yield better clinical outcomes than passive digital tools. Similarly, Bednarski et al. [20] demonstrated that a structured telemedicine program (TeleRecovery) reduced LOS without compromising safety, suggesting that hybrid models combining early discharge with virtual follow-up could optimize resource use. However, the generalizability of these findings may be limited by the small sample size in the study by Bednarski et al. [20], a common challenge in early-phase trials of novel telemedicine applications.

Cost-effectiveness analyses in this review, though limited to two studies, offer valuable insights into the economic viability of telemedicine. Dahlberg et al. [19] found that the RAPP app was cost-effective (mean savings: €23.66 per patient), which aligns with broader evidence that digital follow-up can reduce healthcare costs by minimizing unnecessary visits and complications. However, the absence of significant differences in QALYs underscores the need for longer term evaluations to capture the full economic impact. These observations mirror the conclusions of a Cochrane review by Flodgren et al. [24], which cautioned that while telemedicine can be cost-saving, its long-term value depends on sustained patient engagement and integration into clinical workflows. The feasibility findings of Cremades et al. [21] further highlight implementation challenges, such as lower telemedicine uptake compared to in-person care, which may offset potential cost savings if adoption rates remain suboptimal.

The variability in telemedicine’s impact on clinical outcomes may also stem from differences in surgical complexity and patient risk profiles. For example, studies focusing on minimally invasive or day-case surgeries [17,19] reported more favorable outcomes than those involving major abdominal procedures [10]. This aligns with research by Asiri et al. [25], which suggested that telemedicine is particularly effective for low-risk patients or standardized recovery pathways but may be insufficient for high-risk cohorts requiring intensive monitoring. Additionally, the lack of significant improvement in ERP adherence in Mata et al.'s study [15] raises questions about whether digital tools alone can overcome systemic barriers to compliance, such as patient motivation or institutional protocols. This echoes findings by Ljungqvist et al. [26], who emphasized that technology must complement, not replace, multidisciplinary care coordination to optimize ERP adherence.

Despite the generally low risk of bias across included studies, methodological limitations, such as unblinded designs in the studies by McGillion et al. [14] and Mata et al. [15], introduce some uncertainty in interpreting their findings. The reliance on subjective outcomes (e.g., patient-reported recovery scores) in several studies [11,17,21] further underscores the need for more objective measures in future research. These limitations are consistent with broader critiques of telemedicine RCTs, which often struggle with blinding and outcome standardization, as noted by Dhillon et al. [27]. Nevertheless, the absence of high-risk bias studies in this review strengthens confidence in the overall evidence base.

This systematic review has several limitations. First, the heterogeneity of telemedicine interventions and outcome measures precluded meta-analysis, limiting the ability to quantify pooled effects. Second, most studies had short follow-up periods (e.g., 30 days), which may not capture long-term outcomes or the sustainability of telemedicine benefits. Third, the predominance of high-income country settings (e.g., USA, Canada, Sweden) may limit generalizability to low-resource contexts where telemedicine infrastructure is lacking. Finally, the exclusion of non-RCTs, while methodologically rigorous, may omit insights from real-world implementation studies.

## Conclusions

Telemedicine interventions demonstrate significant potential to enhance postoperative recovery, patient satisfaction, and cost-effectiveness after abdominal surgery, though their impact varies by intervention type and surgical context. While virtual follow-up and digital monitoring tools offer convenient alternatives to traditional care, their success depends on tailored design, patient engagement, and integration into clinical workflows. Future research should prioritize standardized outcome measures, longer term evaluations, and equitable implementation strategies to fully realize telemedicine’s promise in surgical care.

## References

[1] Grygorian A, Montano D, Shojaa M, Ferencak M, Schmitz N (2024). Digital health interventions and patient safety in abdominal surgery: a systematic review and meta-analysis. JAMA Netw Open.

[2] Haveman ME, Jonker LT, Hermens HJ, Tabak M, de Vries JP (2024). Effectiveness of current perioperative telemonitoring on postoperative outcome in patients undergoing major abdominal surgery: a systematic review of controlled trials. J Telemed Telecare.

[3] Parnell K, Kuhlenschmidt K, Madni D (2021). Using telemedicine on an acute care surgery service: improving clinic efficiency and access to care. Surg Endosc.

[4] Williams AM, Bhatti UF, Alam HB, Nikolian VC (2018). The role of telemedicine in postoperative care. Mhealth.

[5] Tenkorang PO, Awuah WA, Mannan KM (2025). The transformative power of telemedicine in delivering effective neurosurgical care in low and middle-income countries: a review. Brain Spine.

[6] Eustache J, El-Kefraoui C, Ekmekjian T, Latimer E, Lee L (2021). Do postoperative telemedicine interventions with a communication feature reduce emergency department visits and readmissions?—a systematic review and meta-analysis. Surg Endosc.

[7] Tham E, Nandra K, Whang SE, Evans NR, Cowan SW (2021). Postoperative telehealth visits reduce emergency department visits and 30-day readmissions in elective thoracic surgery patients. J Healthc Qual.

[8] Page MJ, McKenzie JE, Bossuyt PM (2021). The PRISMA 2020 statement: an updated guideline for reporting systematic reviews. BMJ.

[9] Sterne JA, Savović J, Page MJ (2019). RoB 2: a revised tool for assessing risk of bias in randomised trials. BMJ.

[10] Young JM, Butow PN, Walsh J (2013). Multicenter randomized trial of centralized nurse-led telephone-based care coordination to improve outcomes after surgical resection for colorectal cancer: the CONNECT intervention. J Clin Oncol.

[11] Van Der Meij E, Anema JR, Leclercq WK (2018). Personalised perioperative care by e-health after intermediate-grade abdominal surgery: a multicentre, single-blind, randomised, placebo-controlled trial. Lancet.

[12] Thompson JC, Cichowski SB, Rogers RG (2019). Outpatient visits versus telephone interviews for postoperative care: a randomized controlled trial. Int Urogynecol J.

[13] Pooni A, Brar MS, Anpalagan T (2023). Home to stay: a randomized controlled trial evaluating the effect of a postdischarge mobile app to reduce 30-day readmission following elective colorectal surgery. Ann Surg.

[14] McGillion MH, Parlow J, Borges FK (2021). Post-discharge after surgery Virtual Care with Remote Automated Monitoring-1 (PVC-RAM-1) technology versus standard care: randomised controlled trial. BMJ.

[15] Mata J, Pecorelli N, Kaneva P (2020). A mobile device application (app) to improve adherence to an enhanced recovery program for colorectal surgery: a randomized controlled trial. Surg Endosc.

[16] Lee DD, Arya LA, Andy UU, Harvie HS (2021). Video virtual clinical encounters versus office visits for postoperative care after pelvic organ prolapse surgery: a randomized clinical trial. Female Pelvic Med Reconstr Surg.

[17] Jaensson M, Dahlberg K, Eriksson M, Nilsson U (2017). Evaluation of postoperative recovery in day surgery patients using a mobile phone application: a multicentre randomized trial. Br J Anaesth.

[18] Halder GE, White AB, Brown HW (2022). A telehealth intervention to increase patient preparedness for surgery: a randomized trial. Int Urogynecol J.

[19] Dahlberg K, Philipsson A, Hagberg L, Jaensson M, Hälleberg-Nyman M, Nilsson U (2017). Cost-effectiveness of a systematic e-assessed follow-up of postoperative recovery after day surgery: a multicentre randomized trial. Br J Anaesth.

[20] Bednarski BK, Nickerson TP, You YN (2019). Randomized clinical trial of accelerated enhanced recovery after minimally invasive colorectal cancer surgery (RecoverMI trial). Br J Surg.

[21] Cremades M, Ferret G, Parés D (2020). Telemedicine to follow patients in a general surgery department. A randomized controlled trial. Am J Surg.

[22] Monaghesh E, Hajizadeh A (2020). The role of telehealth during COVID-19 outbreak: a systematic review based on current evidence. BMC Public Health.

[23] Eysenbach G (2001). What is e-health?. J Med Internet Res.

[24] Flodgren G, Rachas A, Farmer AJ, Inzitari M, Shepperd S (2015). Interactive telemedicine: effects on professional practice and health care outcomes. Cochrane Database Syst Rev.

[25] Asiri A, AlBishi S, AlMadani W, ElMetwally A, Househ M (2018). The use of telemedicine in surgical care: a systematic review. Acta Inform Med.

[26] Ljungqvist O, Scott M, Fearon KC (2017). Enhanced recovery after surgery: a review. JAMA Surg.

[27] Dhillon HS, Doermann AC, Walcoff P (1978). Telemedicine and rural primary health care: an analysis of the impact of telecommunications technology. Socio-Econ Plan Sci.

